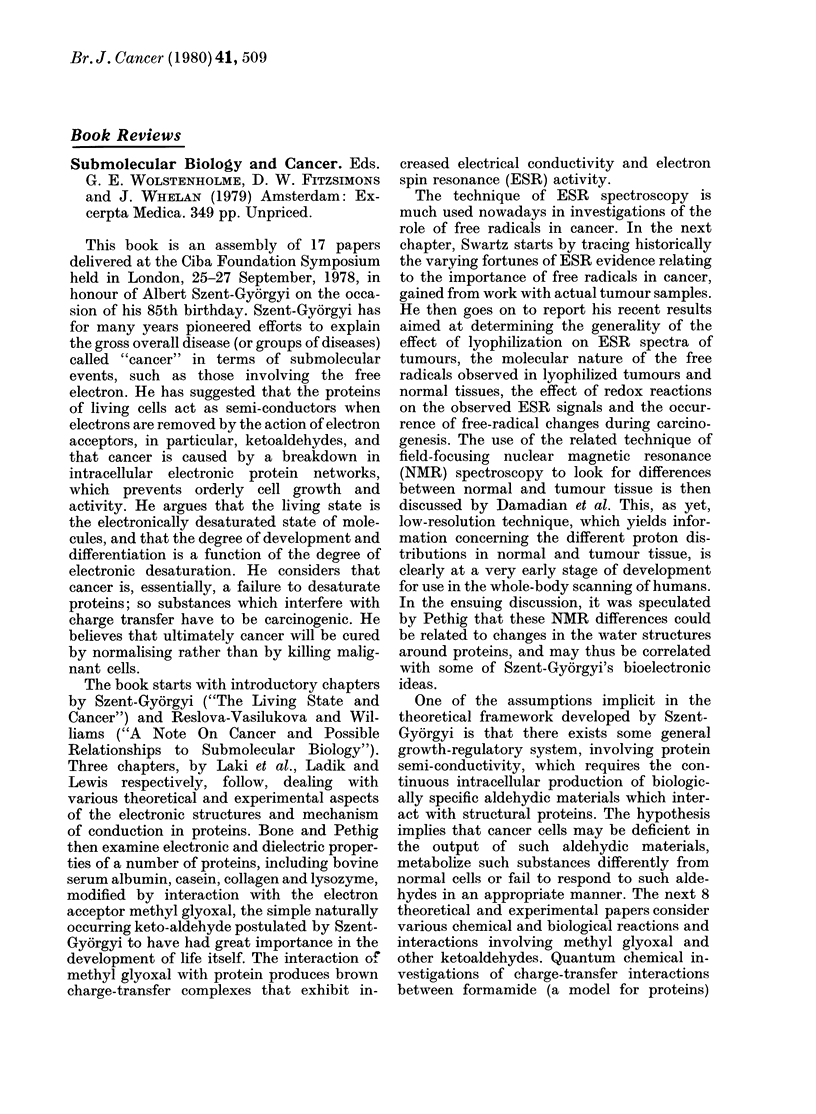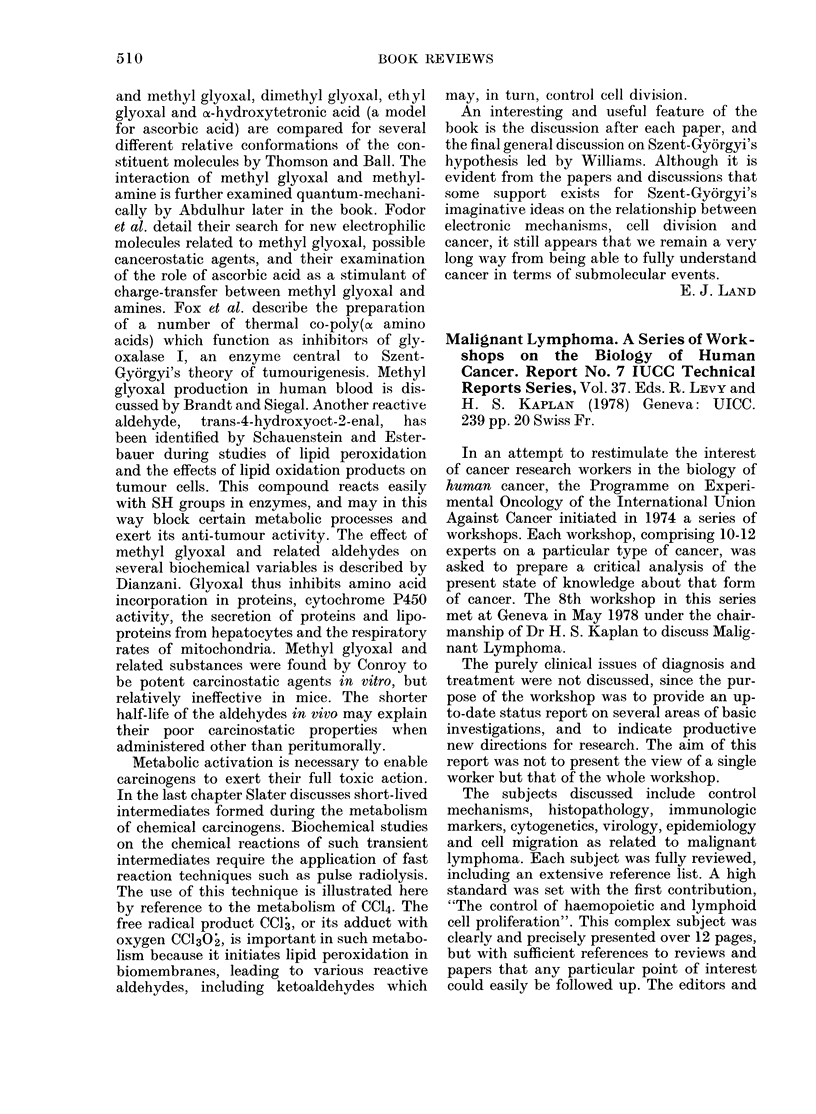# Submolecular Biology and Cancer

**Published:** 1980-03

**Authors:** E. J. Land


*Br. J. Cancer* (1980) **41**, 509

### *Book Reviews*

**Submolecular Biology and Cancer.** Eds. G. E. Wolstenholme, D. W. Fitzsimons and J. Whelan (1979) Amsterdam: Excerpta Medica. 349 pp. Unpriced.

This book is an assembly of 17 papers delivered at the Ciba Foundation Symposium held in London, 25–27 September, 1978, in honour of Albert Szent-Györgyi on the occasion of his 85th birthday. Szent-Györgyi has for many years pioneered efforts to explain the gross overall disease (or groups of diseases) called "cancer" in terms of submolecular events, such as those involving the free electron. He has suggested that the proteins of living cells act as semi-conductors when electrons are removed by the action of electron acceptors, in particular, ketoaldehydes, and that cancer is caused by a breakdown in intracellular electronic protein networks, which prevents orderly cell growth and activity. He argues that the living state is the electronically desaturated state of molecules, and that the degree of development and differentiation is a function of the degree of electronic desaturation. He considers that cancer is, essentially, a failure to desaturate proteins; so substances which interfere with charge transfer have to be carcinogenic. He believes that ultimately cancer will be cured by normalising rather than by killing malignant cells.

The book starts with introductory chapters by Szent-Györgyi ("The Living State and Cancer") and Reslova-Vasilukova and Williams ("A Note On Cancer and Possible Relationships to Submolecular Biology"). Three chapters, by Laki *et al.*, Ladik and Lewis respectively, follow, dealing with various theoretical and experimental aspects of the electronic structures and mechanism of conduction in proteins. Bone and Pethig then examine electronic and dielectric properties of a number of proteins, including bovine serum albumin, casein, collagen and lysozyme, modified by interaction with the electron acceptor methyl glyoxal, the simple naturally occurring keto-aldehyde postulated by Szent-Györgyi to have had great importance in the development of life itself. The interaction of methyl glyoxal with protein produces brown charge-transfer complexes that exhibit increased electrical conductivity and electron spin resonance (ESR) activity.

The technique of ESR spectroscopy is much used nowadays in investigations of the role of free radicals in cancer. In the next chapter, Swartz starts by tracing historically the varying fortunes of ESR evidence relating to the importance of free radicals in cancer, gained from work with actual tumour samples. He then goes on to report his recent results aimed at determining the generality of the effect of lyophilization on ESR spectra of tumours, the molecular nature of the free radicals observed in lyophilized tumours and normal tissues, the effect of redox reactions on the observed ESR signals and the occurrence of free-radical changes during carcinogenesis. The use of the related technique of field-focusing nuclear magnetic resonance (NMR) spectroscopy to look for differences between normal and tumour tissue is then discussed by Damadian *et al.* This, as yet, low-resolution technique, which yields information concerning the different proton distributions in normal and tumour tissue, is clearly at a very early stage of development for use in the whole-body scanning of humans. In the ensuing discussion, it was speculated by Pethig that these NMR differences could be related to changes in the water structures around proteins, and may thus be correlated with some of Szent-Györgyi's bioelectronic ideas.

One of the assumptions implicit in the theoretical framework developed by Szent-Györgyi is that there exists some general growth-regulatory system, involving protein semi-conductivity, which requires the continuous intracellular production of biologically specific aldehydic materials which interact with structural proteins. The hypothesis implies that cancer cells may be deficient in the output of such aldehydic materials, metabolize such substances differently from normal cells or fail to respond to such aldehydes in an appropriate manner. The next 8 theoretical and experimental papers consider various chemical and biological reactions and interactions involving methyl glyoxal and other ketoaldehydes. Quantum chemical investigations of charge-transfer interactions between formamide (a model for proteins)

and methyl glyoxal, dimethyl glyoxal, ethyl glyoxal and α-hydroxytetronic acid (a model for ascorbic acid) are compared for several different relative conformations of the constituent molecules by Thomson and Ball. The interaction of methyl glyoxal and methylamine is further examined quantum-mechanically by Abdulhur later in the book. Fodor *et al.* detail their search for new electrophilic molecules related to methyl glyoxal, possible cancerostatic agents, and their examination of the role of ascorbic acid as a stimulant of charge-transfer between methyl glyoxal and amines. Fox *et al.* describe the preparation of a number of thermal co-poly(α amino acids) which function as inhibitors of glyoxalase I, an enzyme central to Szent-Györgyi's theory of tumourigenesis. Methyl glyoxal production in human blood is discussed by Brandt and Siegal. Another reactive aldehyde, trans-4-hydroxyoct-2-enal, has been identified by Schauenstein and Esterbauer during studies of lipid peroxidation and the effects of lipid oxidation products on tumour cells. This compound reacts easily with SH groups in enzymes, and may in this way block certain metabolic processes and exert its anti-tumour activity. The effect of methyl glyoxal and related aldehydes on several biochemical variables is described by Dianzani. Glyoxal thus inhibits amino acid incorporation in proteins, cytochrome P450 activity, the secretion of proteins and lipoproteins from hepatocytes and the respiratory rates of mitochondria. Methyl glyoxal and related substances were found by Conroy to be potent carcinostatic agents *in vitro*, but relatively ineffective in mice. The shorter half-life of the aldehydes *in vivo* may explain their poor carcinostatic properties when administered other than peritumorally.

Metabolic activation is necessary to enable carcinogens to exert their full toxic action. In the last chapter Slater discusses short-lived intermediates formed during the metabolism of chemical carcinogens. Biochemical studies on the chemical reactions of such transient intermediates require the application of fast reaction techniques such as pulse radiolysis. The use of this technique is illustrated here by reference to the metabolism of $CCl_4$. The free radical product $CCl_3^{\cdot}$, or its adduct with oxygen $CCl_3O_2^{\cdot}$, is important in such metabolism because it initiates lipid peroxidation in biomembranes, leading to various reactive aldehydes, including ketoaldehydes which may, in turn, control cell division.

An interesting and useful feature of the book is the discussion after each paper, and the final general discussion on Szent-Györgyi's hypothesis led by Williams. Although it is evident from the papers and discussions that some support exists for Szent-Györgyi's imaginative ideas on the relationship between electronic mechanisms, cell division and cancer, it still appears that we remain a very long way from being able to fully understand cancer in terms of submolecular events.

E. J. Land